# Preliminary development and internal evaluation of a plasma *SOX1* methylation-based model bladder cancer detection

**DOI:** 10.1515/biol-2025-1396

**Published:** 2026-11-04

**Authors:** Guangtao Zhang, Jiangning Mu, Xuezhen Shi, Zhenhu Bao, Yonghong Ma, Li Kong, Lin Zhao, Dingming Bai, Yuyong Ding, Peng Du, Fei Dong

**Affiliations:** Department of Urology, The Second People’s Hospital of Shizuishan, Shizuishan, 753000, Ningxia Hui Autonomous Region, China; The First Clinical Medical College of Ningxia Medical University, Yinchuan, 750004, Ningxia Hui Autonomous Region, China

**Keywords:** bladder cancer, DNA methylation, diagnostic model, liquid biopsy, *SOX1*

## Abstract

The evaluation of individuals with suspected bladder cancer (BCa) continues to rely on cystoscopy, while urine cytology demonstrates limited sensitivity. This single-center retrospective two-gate case-control study included 90 individuals (30 with BCa and 60 non-cancer controls). *SOX1* methylation signals in plasma-derived DNA were quantified using quantitative methylation-specific PCR. Logistic regression was used to generate a *SOX1*-only model and a combined model incorporating *SOX1* methylation, age, and sex. Model performance was described using apparent ROC-AUC, sensitivity, specificity and other operating characteristics at the Youden-derived cut-off, together with internal bootstrap, five-fold cross-validation, calibration, and exploratory decision-curve analysis (DCA). All resampling procedures used the same 90 participants; no independent validation cohort was available. *PAX1* methylation was assessed as a prespecified exploratory single-marker comparator. In this selected sample, the combined model had a higher apparent AUC than the *SOX1*-only model (0.882 vs 0.828). At the Youden-derived threshold, sensitivity was 83.3 % and specificity was 88.3 %. Exploratory DCA favored the combined model within the study sample, but this estimate is dependent on the fixed case-control sampling fraction. The *PAX1-*only AUC methylation was 0.845, numerically higher than the *SOX1*-only AUC, although *PAX1* was not included in multivariable modeling. Adding age and sex to the plasma *SOX1* methylation signal improved apparent discrimination within this small selected cohort. This preliminary, hypothesis-generating model is not validated for clinical use and should not be assumed to generalize to consecutive hematuria populations. Independent prospective evaluation in larger, clinically representative cohorts is required.

## 1 Introduction

Bladder cancer (BCa) is among the most prevalent malignancies of the urinary system worldwide. According to 2023 data from the International Agency for Research on Cancer (IARC) of World Health Organization (WHO), the global annual incidence of BCa is approximately 573,000 new cases, with an estimated 213,000 deaths each year [1], [2], [3], [4], [5]. In China, BCa represents the most common urological malignancy, and its incidence continues to increase. Prognosis is strongly dependent on the stage at diagnosis: patients with early-stage disease (Ta–T1) demonstrate a 5-year survival rate exceeding 90 %, whereas survival decreases to 30–40 % in those with advanced-stage disease (T2–T4) [2], [6], [7], [8], [9]. Therefore, early and accurate detection remains critical for improving outcomes.

Cystoscopy with pathological confirmation remains the clinical gold standard; however, it is invasive, costly, and unsuitable for large-scale screening [10], 11]. Urine cytology is non-invasive but demonstrates limited sensitivity (often 40–60 %), particularly in individuals with low-grade tumors [12]. Therefore, there is sustained interest in molecular biomarker–based diagnostic strategies that may reduce reliance on invasive procedures while maintaining acceptable diagnostic accuracy.

Aberrant deoxyribonucleic acid (DNA) methylation represents an early epigenetic event in carcinogenesis and exhibits properties favorable for liquid biopsy–based diagnostics, including tissue specificity, stability, and detectability in plasma and urine [13], [14], [15], [16], [17]. SRY-related high-mobility group box (HMG-box) gene 1 (*SOX1*), a member of the SOX transcription factor family, is involved in embryonic development and differentiation. Promoter hypermethylation of *SOX1* may result in gene silencing and has been reported across multiple malignancies, supporting its potential as a diagnostic biomarker [18], [19], [20], [21]. Paired box 1 (*PAX1*) is another tumor suppressor gene frequently silenced through promoter hypermethylation and has demonstrated diagnostic potential in other tumor settings; however, evidence supporting its use in blood-based assays for BCa remains limited.

A preliminary diagnostic model for BCa was developed and internally evaluated by integrating plasma *SOX1* methylation signals with basic clinical variables (age and sex). The performance of the combined model was assessed and compared with that of a *SOX1*-only model, and plasma *PAX1* methylation was assessed as an exploratory single-marker comparator. The aim was to examine feasibility and generate internally derived performance estimates; the study was not designed to establish a clinically validated test or generalizability.

## 2 Methods

### 2.1 Study design

A single-center retrospective two-gate case-control diagnostic test study was conducted in accordance with the Declaration of Helsinki. The present study used only existing residual blood specimens that had originally been collected for routine clinical care or health examinations; no additional blood samples were collected specifically for this research.

**Informed consent:** Informed consent was obtained from all individuals participating in this study, or from their legal guardians or wards.

**Ethical approval:** The research related to human use has been complied with all the relevant national regulations, institutional policies and in accordance with the tenets of the Helsinki Declaration, and has been approved by the Ethics Committee of The Second People’s Hospital of Shizuishan, China (approval No. 201902510-2).

### 2.2 Study participants

A total of 120 eligible individuals were screened from the institutional database between January 2019 and December 2023. 90 individuals (30 patients with BCa and 60 non-cancer controls) were included in the primary analysis cohort for preliminary model development and internal evaluation. 30 individuals presenting for bladder cancer re-examination were excluded from the primary cohort to maintain a clear case definition for initial diagnosis; these individuals were assessed only in exploratory analyses. All resampling-based evaluations used the same 90 participants; no independent external validation cohort was available.

### 2.3 Inclusion criteria

BCa group: (i) newly diagnosed urothelial carcinoma in the Department of Urology, confirmed by pathological examination of cystoscopic biopsy or surgical resection specimens in accordance with the 2022 WHO Classification of Tumors of the Urinary System; (ii) availability of residual peripheral blood originally collected within one week prior to surgery as part of routine clinical care; (iii) availability of complete clinical data.

Non-cancer control group: (i) individuals from the Health Examination Center with no history of urinary system disease and with normal urinalysis and urinary ultrasound findings; (ii) individuals with benign urological conditions (including benign prostatic hyperplasia, renal stones, and urinary tract infections), confirmed by clinical and imaging examinations with malignancy excluded; (iii) blood sampling time aligned with that of the BCa group; (iv) availability of complete clinical data. For Health Examination Center participants, the samples used in this study were residual material remaining after routine health examination testing; no additional tube was collected for research. Samples from the benign urological control subgroup likewise originated from routine clinical testing.

### 2.4 Exclusion criteria

For all individuals: (i) history of other malignancies; (ii) severe hepatic or renal dysfunction (Child–Pugh class C) or acute infectious diseases; (iii) absence of pathological confirmation (for individuals with bladder cancer) or lack of clinical assessment or follow-up documentation after blood collection (for control individuals); (iv) missing key variables (age, sex, or diagnostic outcome).

### 2.5 Sample collection and laboratory analysis

#### 2.5.1 Blood sample processing

All blood specimens used in this study were residual samples originally obtained for routine clinical care or health examinations, rather than for the present research. No additional venipuncture or research-specific blood collection was performed. The source peripheral venous blood specimens (5 mL) had originally been collected after an overnight fast into dipotassium ethylenediaminetetraacetic acid (EDTA-K2) tubes. Plasma was separated by centrifugation at 3,000 rpm for 10 min at 4 °C within 1 h of original collection and stored at −80 °C until DNA extraction. Residual specimens and their associated research data were anonymized or coded before secondary research use.

#### 2.5.2 Quantification of *SOX1* and *PAX1* methylation signals

Plasma *SOX1* and *PAX1* methylation signals were quantified using quantitative methylation-specific PCR (qMSP).

Plasma-derived DNA extraction: cell-free DNA (cfDNA) was extracted from 1–2 mL of plasma using the QIAamp Circulating Nucleic Acid Kit (Qiagen, Germany). DNA concentration and purity were assessed using a Nanodrop 2000 spectrophotometer (Thermo Fisher, USA); samples with an A260/A280 ratio between 1.8 and 2.0 were considered acceptable. Because the protocol did not fractionate freely circulating DNA, extracellular-vesicle-associated DNA, or residual cell-derived DNA, the recovered material is referred to as plasma-derived DNA rather than cfDNA in this study.

Bisulfite conversion: Plasma-derived DNA was subjected to bisulfite conversion using the EpiTect Bisulfite Kit (Qiagen, Germany), whereby unmethylated cytosines were converted to uracil, while methylated cytosines remained unchanged.

qMSP amplification and quantification: Reactions were performed in a total volume of 20 μL, comprising 10 μL of 2× Taq polymerase chain reaction (PCR) MasterMix, 0.5 μL each of forward and reverse primers, 2 μL of bisulfite-modified DNA, and 7 μL of nuclease-free water. Primer sequences were as follows:

*SOX1*-Methylated: Forward 5′-GTT​TTA​GAG​GGT​TAT​CGC​GT-3′, Reverse 5′-CAA​ACG​AAA​AAC​CGC​GAA-3′

*PAX1*-Methylated: Forward 5′-TGA​CCA​TGA​ACC​ATG​AAG​GA-3′, Reverse 5′-GAT​TTA​GTG​GGT​CAT​CAA​GT-3′

Glyceraldehyde-3-phosphate dehydrogenase (GAPDH) (Reference): Forward 5′-TGG​GTG​TGA​ACC​ATG​AGA​AG-3′, Reverse 5′-GAG​TCC​TTC​CAC​GAT​ACC​AA-3′

Amplification was conducted on an ABI 7500 Real-Time PCR System (Thermo Fisher, USA) under the following conditions: initial denaturation at 95 °C for 5 min; 40 cycles of 95 °C for 30 s, 58 °C for 30 s, and 72 °C for 30 s; followed by a final extension at 72 °C for 10 min. Each sample was tested in triplicate, and the results were averaged.

### 2.6 Quantification metric

A standard curve was constructed using serially diluted methylated control DNA. The methylation “signal” was calculated as 2^(−ΔCt) × 100, where ΔCt = Ct(target) − Ct (GAPDH). As this calculation yields a relative signal (normalized to GAPDH) rather than an absolute biochemical methylation percentage, it was referred to throughout the manuscript as a “relative methylation signal”.

### 2.7 Statistical analysis

Analyses were conducted using R (v4.3.1). A two-sided *p* value < 0.05 was considered statistically significant for descriptive comparisons. For the prespecified model comparison (combined model vs *SOX1*-only), a one-sided DeLong test was applied under the directional alternative that the combined model improved discrimination.

Continuous variables were summarized as mean ± standard deviation (SD) or median (interquartile range (IQR)), as appropriate, and compared using t-tests (or analysis of variance (ANOVA), where applicable). Categorical variables were summarized as *n* (%) and compared using chi-square or Fisher’s exact tests.

No formal *a priori* sample-size calculation was performed; the sample size was determined by the availability of eligible retrospective records and specimens. The combined model contained three predictor parameters but only 30 cancer events. Coefficient instability, overfitting, and optimistic performance estimates are therefore substantial concerns, and all model analyses were regarded as preliminary and hypothesis-generating.

### 2.8 Model development and internal evaluation

Binary logistic regression models were fitted: Model 1 (*SOX1*-only) and Model 2 (combined: *SOX1* + age + sex). *PAX1* was fitted as a prespecified exploratory single-marker comparator and was not added to the multivariable model. This avoided post hoc marker selection and further model expansion after inspection of the same small development sample; it does not imply that *SOX1* was superior to *PAX1*.

Discrimination: Apparent area under the curves (AUCs) and 95 % confidence intervals (CIs) were estimated in the full cohort using bootstrap resampling (5,000 resamples). Bootstrap resampling was an internal procedure and did not provide an independent validation estimate.

Stability: Five-fold stratified cross-validation was performed using the same 90 participants. Each fold contained approximately 6 cases and 12 controls; in each iteration, the model was refitted in four folds and evaluated in the remaining fold, and fold-specific AUCs were averaged. This procedure was interpreted only as an internal stability assessment.

Model comparison: The difference in AUC (ΔAUC) between Model 1 and Model 2 was assessed using DeLong’s test for correlated receiver operating characteristic (ROC) curves (one-sided). The 95 % CI for ΔAUC was also obtained using paired bootstrap resampling (5,000 resamples). All comparisons were based on the same development cohort.

Clinical operating characteristics: The optimal cut-off was determined using the Youden index. Sensitivity, specificity, accuracy, positive predictive value (PPV), and negative predictive value (NPV) were reported at the selected threshold. Because the study used a fixed 1:2 case-control sampling fraction, PPV, NPV, and accuracy were reported descriptively and are not population-level estimates.

Exploratory decision-curve analysis (DCA) was used to assess net benefit across threshold probabilities. Because no representative clinical cohort was available, the net-benefit results were interpreted as sample-dependent and not as evidence of clinical utility.

### 2.9 Visualization and calibration

ROC curves were generated for Model 1 and Model 2. Calibration for Model 2 was assessed using bootstrap resampling (*B* = 200) and was visualized using apparent and bias-corrected calibration curves. All calibration analyses were based on the same 90 participants and were interpreted cautiously because of the small sample size and selected case-control design.

## 3 Results

### 3.1 Baseline characteristics

Baseline characteristics of the model development cohort (*n* = 90) are presented in Table 1. The cohort comprised 30 individuals with BCa and 60 non-cancer controls (30 healthy individuals and 30 individuals with benign urological conditions). The cohort was predominantly male (78.9 %) with a mean age of 61.86 ± 13.65 years. Differences in sex distribution and age were observed between the BCa and control groups (*p* = 0.004 and *p* = 0.002, respectively). Among the 30 BCa cases, 29 (96.7 %) were male. 12 patients (40.0 %) had Ta/Tis disease, 12 (40.0 %) had T1 disease, and 6 (20.0 %) had ≥T2 disease; 12 (40.0 %) tumors were low grade and 18 (60.0 %) were high grade (Table 1). These characteristics are reported descriptively because the subgroup sizes were insufficient for stable stage- or grade-specific performance analyses.

**Table 1:** Baseline characteristics of the model development cohort (*n* = 90).

Variable	Overall (*n* = 90)	Bladder cancer (*n* = 30)	Non-cancer controls (*n* = 60)	*p*
Male, *n* (%)	71 (78.9)	29 (96.7)	42 (70.0)	0.004
Age (years), mean ± SD	61.86 ± 13.65	68.97 ± 13.18	58.67 ± 13.38	0.002
Tumor stage, *n* (%)	–		–	–
Ta/Tis	–	12 (40.0)	–	–
T1	–	12 (40.0)	–	–
≥T2	–	6 (20.0)	–	–
Tumor grade, *n* (%)	–		–	–
Low grade	–	12 (40.0)	–	–
High grade	–	18 (60.0)	–	–

Continuous variables are presented as mean ± standard deviation. Categorical variables are presented as number (%). Tumor stage and grade are applicable only to patients with bladder cancer and are presented descriptively; between-group statistical comparisons were not applicable.

### 3.2 *SOX1* and *PAX1* relative methylation signals

Relative methylation signals of *SOX1* and *PAX1* are summarized in Table 2. Both markers differed significantly between individuals with BCa and control individuals (both *p* < 0.001). Within the control group, no statistically significant difference was detected between healthy individuals and individuals with benign urological conditions (*p* > 0.05); these subgroups were pooled for the preliminary analysis, but this finding does not establish equivalence or representative clinical specificity.

**Table 2:** *SOX1* and *PAX1* relative methylation signals in the model development cohort.

Marker	Bladder cancer (*n* = 30) Median (IQR)	Non-cancer controls (*n* = 60) Median (IQR)	*p*
*SOX1* signal (2^(−ΔCt) × 100)	85.15 (30.25)	12.35 (35.65)	<0.001
*PAX1* signal (2^(−ΔCt) × 100)	90.45 (25.50)	18.74 (45.65)	<0.001

IQR, interquartile range. *PAX1* was evaluated in an exploratory fashion; *SOX1* was the prespecified primary biomarker for model construction.

### 3.3 Model parameters

Logistic regression parameters are presented in Table 3. In the combined model, higher *SOX1* methylation signal and older age were significantly associated with bladder cancer, whereas sex did not reach statistical significance. The wide CI for sex reflected substantial imprecision in this sex-imbalanced sample.

**Table 3:** Parameters of the diagnostic logistic regression models.

Model/predictor	*β*	OR	95 % CI for OR	*p*
Intercept (*SOX1*-only)	−2.451	–	–	<0.001
*SOX1* (*SOX1*-only)	0.078	1.081	1.044–1.120	<0.001
Intercept (combined)	−9.874	–	–	<0.001
*SOX1* (combined)	0.065	1.067	1.030–1.106	<0.001
Age (combined)	0.063	1.065	1.013–1.120	0.012
Sex, male (combined)	1.843	6.319	0.563–70.923	0.134

### 3.4 Model performance

Model performance is summarized in Table 4; all values are apparent estimates from the selected development sample. The combined model had a higher AUC than the *SOX1*-only model (AUC 0.882 vs 0.828), with an AUC difference of 0.053 (one-sided *p* = 0.043 by DeLong test under the prespecified directional hypothesis). This modest within-sample improvement should be interpreted cautiously. At the selected threshold, sensitivity was 83.3 % and specificity was 88.3 % for the combined model. The exploratory *PAX1*-only model had an AUC of 0.845, numerically higher than the *SOX1*-only AUC of 0.828; no formal superiority comparison between the two single markers was planned.

**Table 4:** Apparent diagnostic performance of models for bladder cancer detection.

Model	AUC (95 % CI)	Sensitivity (%)	Specificity (%)	PPV (%)	NPV (%)	Accuracy (%)
*SOX1*-only	0.828 (0.716–0.922)	86.7	83.3	72.2	92.6	84.4
*PAX1*-only (exploratory)	0.845 (0.735–0.935)	83.3	85.0	75.0	91.1	84.4
Combined (*SOX1* + age + sex)	0.882 (0.800–0.948)	83.3	88.3	78.6	91.5	86.7

AUC, area under the ROC curve; CI, confidence interval; PPV, positive predictive value; NPV, negative predictive value. All values are apparent estimates from the same selected case-control sample. PPV, NPV, accuracy, calibration, and DCA depend on the fixed 1:2 case-control sampling fraction and should not be interpreted as population-level clinical performance.

### 3.5 Exploratory DCA

Within the selected study sample, exploratory DCA favored the combined model over the *SOX1*-only model and the treat-all/treat-none strategies across threshold probabilities of approximately 10–70 %. Because net benefit was calculated using the fixed 1:2 case-control sampling fraction and no representative cohort was available, this finding does not establish real-world clinical utility.

### 3.6 Exploratory analysis across broader clinical groups

In exploratory analyses including additional clinical groups (e.g., BCa re-examination, benign prostatic hyperplasia, and prostate cancer), *SOX1* and *PAX1* methylation signals were elevated compared with those observed in healthy individuals (Figure 1). These findings do not establish BCa specificity and may reflect cancer-associated or other nonspecific sources of plasma DNA methylation. The exploratory groups were not used in model development or formal specificity evaluation.

**Figure 1:**
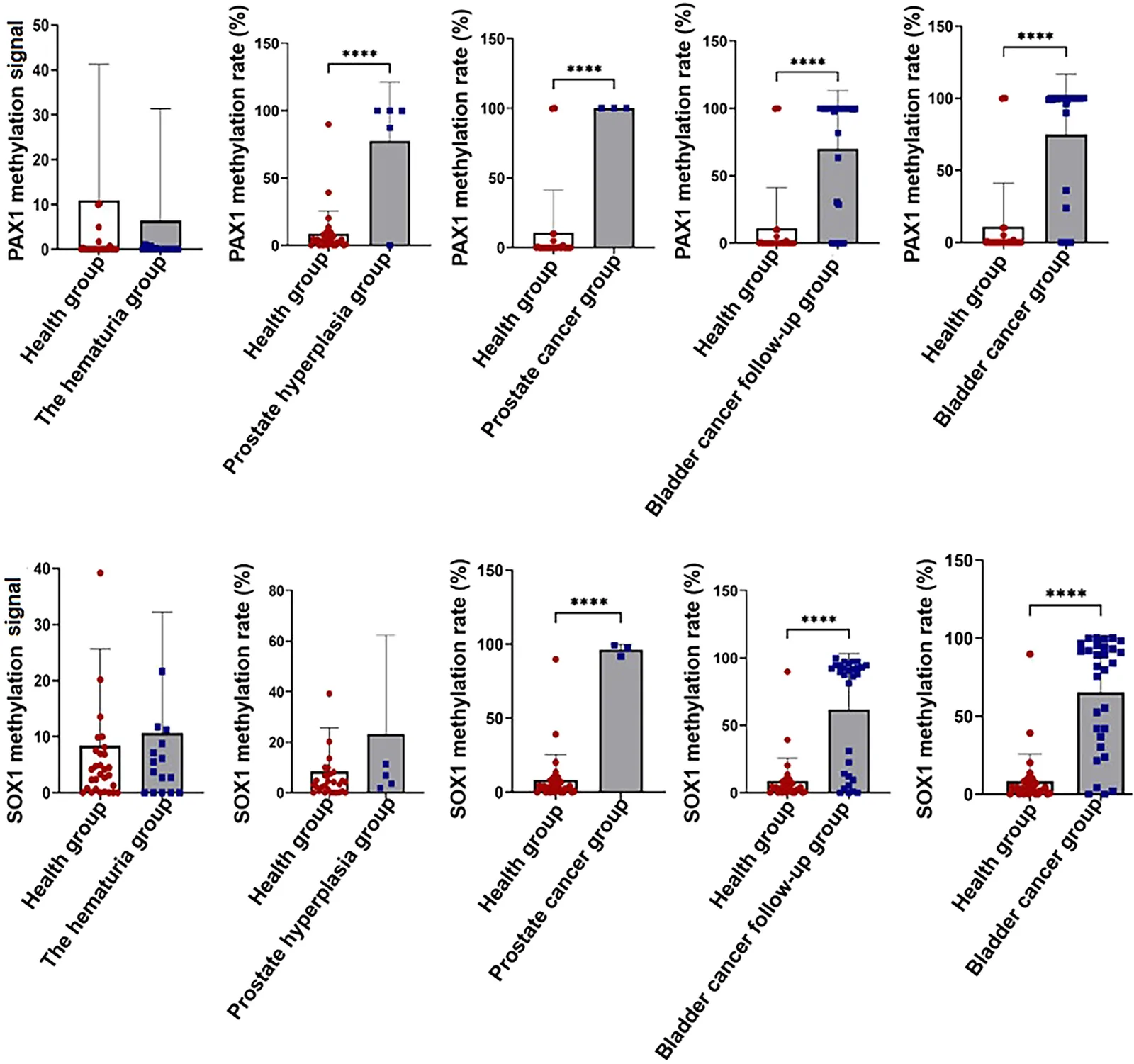
Distribution of *SOX1* and *PAX1* methylation signals across different clinical groups. *Y*-axis values represent the relative qMSP signal (2^(−ΔCt) × 100) and not an absolute methylation percentage. These exploratory groups were not included in model development.

### 3.7 Visual analysis

#### 3.7.1 Visual assessment of discrimination and calibration

Figure 2 presents ROC curves for the *SOX1*-only and combined models, consistent with the apparent AUC values reported in Table 4 (*SOX1*-only AUC 0.828; combined AUC 0.882). Figure 3 shows that the apparent calibration curve was closer to the ideal line, whereas the bias-corrected curve was irregular, particularly at the extremes. This instability is consistent with the small sample and should not be interpreted as independent validation.

**Figure 2:**
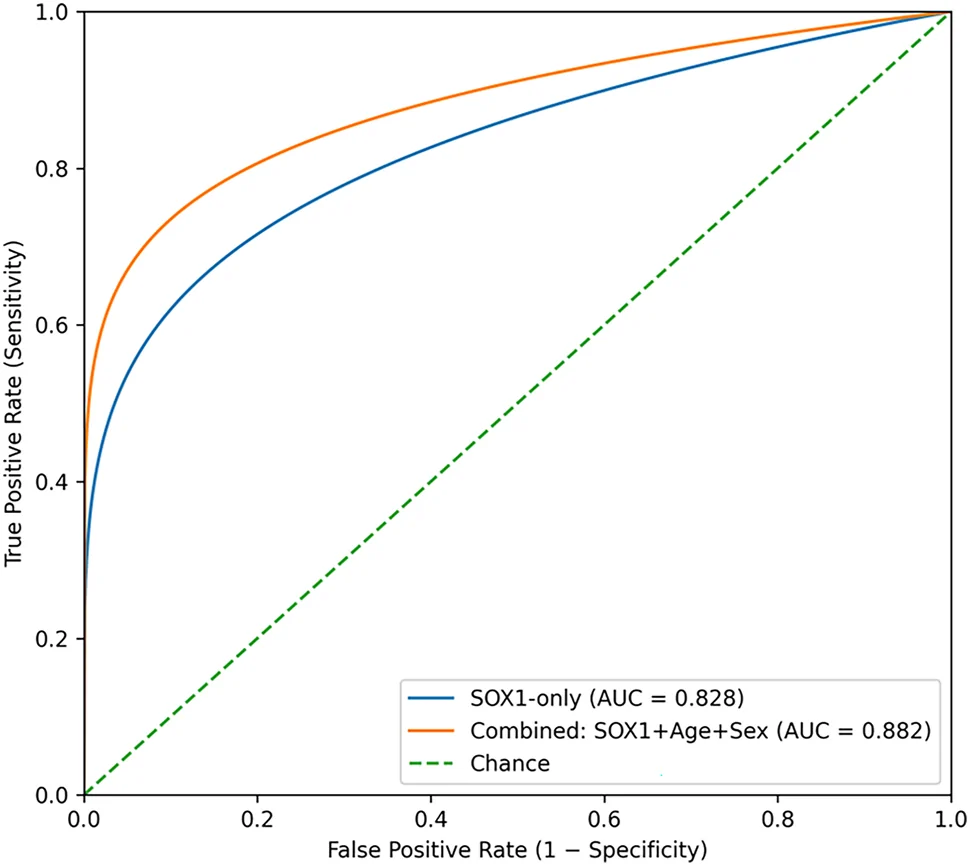
Comparison of ROC curves for the diagnostic models. ROC curves for the *SOX1*-only model and the combined model (*SOX1* + age + sex) are shown. Values are apparent AUCs from the selected development sample (*SOX1*-only AUC = 0.828; combined AUC = 0.882).

**Figure 3:**
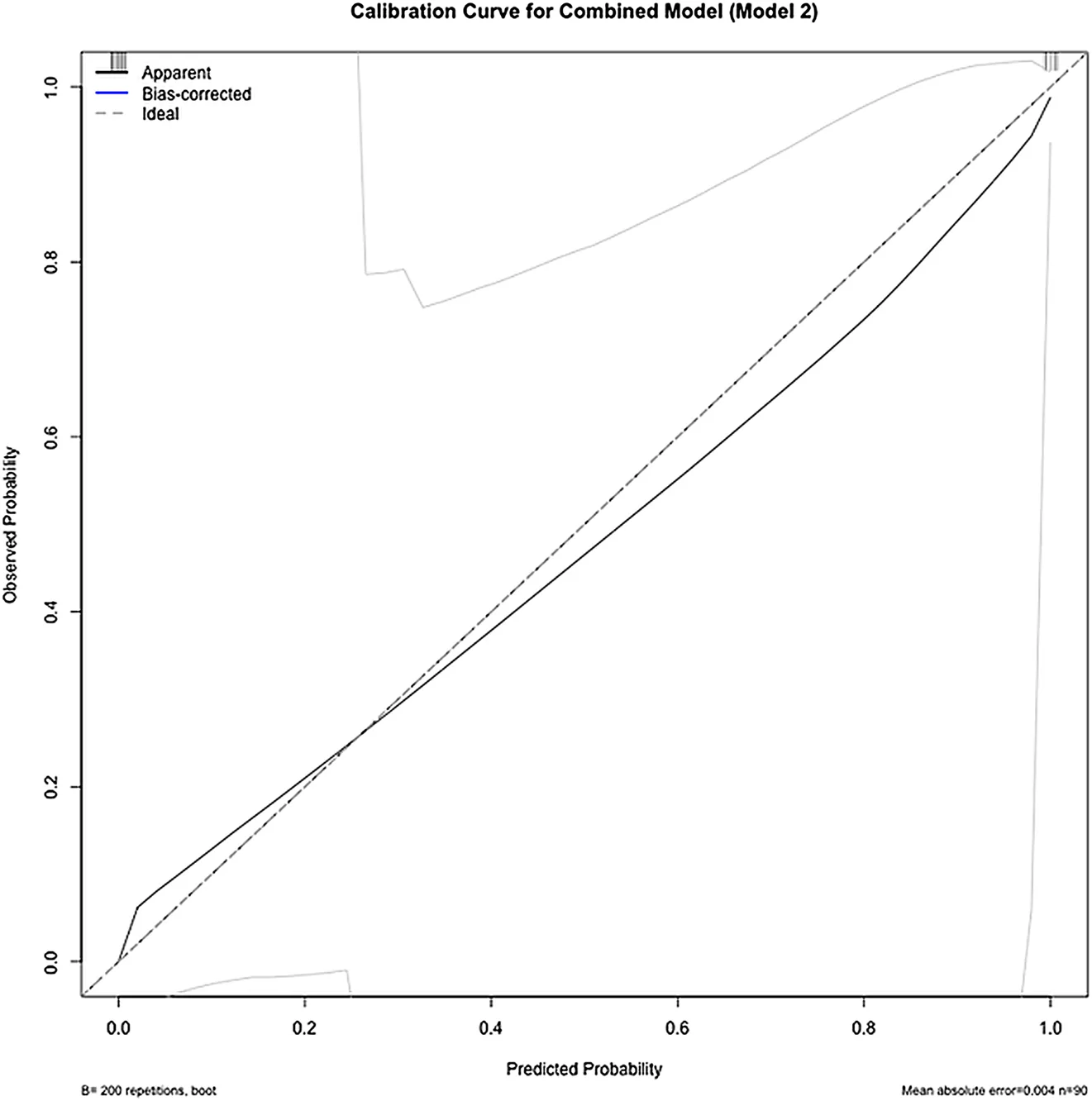
Calibration curve for the combined diagnostic model (*SOX1* + age + sex). Calibration was assessed via bootstrapping (*B* = 200) using the same cohort. The apparent and bias-corrected curves are shown with the ideal diagonal reference.

## 4 Discussion

In this single-center retrospective two-gate case-control study, a preliminary plasma *SOX1* methylation–based model for BCa detection was developed and internally evaluated. Three observations warrant cautious consideration. First, *SOX1* and *PAX1* relative methylation signals measured in plasma-derived DNA were significantly higher in individuals with newly diagnosed BCa cases than in the selected non-cancer controls, including benign urological controls Second, within this development sample, adding age and sex to *SOX1* increased the apparent AUC from 0.828 to 0.882 and yielded sensitivity of 83.3 % and specificity of 88.3 % at the Youden-derived cut-off; however, the small event count, sex imbalance, and irregular bias-corrected calibration curve indicate substantial uncertainty and possible optimism. Third, the *PAX1*-only AUC (0.845) was numerically higher than the *SOX1*-only AUC, and both markers were elevated in other clinical groups, so BCa specificity is not established. These findings are hypothesis-generating and do not constitute clinical validation.

Clinical pathways for individuals with suspected BCa rely predominantly based on cystoscopy and cross-sectional imaging, which are invasive, resource-intensive, and variably tolerated. Hematuria, particularly microhematuria, is a common indication for referral and further evaluation; however, the diagnostic yield for malignancy is modest in many real-world settings [22], 23]. Contemporary American Urological Association/Society of Urodynamics, Female Pelvic Medicine & Urogenital Reconstruction (AUA/SUFU) microhematuria guidelines therefore emphasize risk stratification using patient factors, including age and sex, to tailor the intensity of evaluation and to reduce unnecessary testing in low-risk individuals [24], 25].

In principle, a blood-based assay could complement guideline-directed risk assessment. However, the present selected case-control study cannot establish a triage role, a reduction in cystoscopy, or any safe clinical operating threshold. Such claims require prospective evaluation in consecutive hematuria populations, with direct comparison against current diagnostic pathways and emphasis on clinically significant disease.

The biological rationale for evaluating plasma DNA methylation is supported by prior cfDNA literature. DNA methylation changes represent early and widespread events in carcinogenesis, and can be amplified using PCR-based approaches following bisulfite conversion, and may yield a higher signal-to-noise ratio than somatic mutations in settings with low tumor fraction. Large multi-cancer studies have demonstrated that targeted methylation profiling of plasma cfDNA can detect cancer-associated signals with high specificity and may assist in tissue-of-origin localization, providing proof-of-concept for blood-based methylation detection [26]. However, translation of methylation assays from research settings to routine clinical practice requires careful consideration of pre-analytical and analytical variability, including DNA extraction yield, bisulfite conversion efficiency, storage conditions, and contamination by leukocyte-derived DNA contamination.

Recent consensus-oriented guidance emphasizes the need for standardized blood processing, quality-control measures, and transparent reporting of pre-analytical variables for methylation-based liquid biopsies [27]. The workflow in this study included plasma separation within 1 h, storage at −80 °C, and qMSP quantification normalized to a reference gene. Nevertheless, a single centrifugation step was used and no DNA fractionation was performed; therefore, the study material cannot be assumed to consist exclusively of cfDNA. Further pre-analytical standardization, inter-laboratory harmonization, and external analytical validation are needed before clinical implementation.

Although this study focused on plasma, urinary methylation assays provide an important benchmark, as urine is in direct contact with urothelial tumors and often contains a higher fraction of tumor-derived DNA. An increasing body of evidence supports the diagnostic potential of urinary methylation markers, including qMSP-based panels that achieve AUCs in the high 0.80 s in prospective or preclinical validation settings [28]. A 2024 systematic review and meta-analysis synthesizing a large body of literature reported that urinary DNA methylation biomarkers can achieve high diagnostic accuracy for both primary and recurrent BCa, while also highlighting substantial heterogeneity and a lack of large validation studies in clinically representative populations [29].

More recently, a urinary DNA methylation test assessed for hematuria-associated BCa diagnosis in a multicenter setting further demonstrated that methylation-based biomarkers can be evaluated within real-world diagnostic pathways and assessed using clinically meaningful endpoints, including decision-analytic measures [30]. Collectively, these findings indicate that methylation biomarkers are approaching clinical applicability; however, they also underscore a key translational consideration: performance estimates derived from case–control designs may be inflated compared with those observed in consecutive hematuria cohorts, in which benign inflammatory conditions, urolithiasis, prior instrumentation, and other malignancies contribute to background variability against which specificity must be maintained.

A plasma-based assay may offer practical advantages despite the biological proximity of urine to the tumor. Blood collection is standardized, can be performed when urine samples are unavailable or contaminated, and may better reflect systemic tumor burden or occult advanced disease. Plasma-based testing may integrate with other blood-based evaluations in individuals undergoing assessment for hematuria and may support longitudinal monitoring when urinary tumor DNA shedding is intermittent. However, tumor-derived DNA signals in blood are often attenuated in individuals with early-stage or low-grade disease, and this limitation is well recognized in the broader liquid biopsy literature for bladder cancer.

Recent high-level reviews indicate that circulating tumor DNA (ctDNA) and circulating tumor cells (CTCs) represent promising but technically challenging biomarkers; assay selection, tumor fraction, and stage distribution strongly influence detectability, and clinical implementation requires rigorous prospective trials and fit-for-purpose workflows [31], [32], [33]. The present findings show only that *SOX1* and *PAX1* methylation signals can be measured in plasma-derived DNA and can separate selected groups in a small cohort; they do not identify the DNA source or establish BCa-specific diagnostic validity.

From a modeling perspective, the incremental value of incorporating age and sex with *SOX1* methylation is clinically plausible. Age is a well-established predictor of urothelial malignancy and is incorporated into guideline-based risk stratification algorithms for hematuria [24], 25]. In the multivariable model, older age and higher *SOX1* methylation signal were independently associated with BCa, whereas sex did not reach statistical significance. likely reflecting the limited sample size, imbalance in sex distribution, and wide CIs typical of small datasets. With 29 of 30 BCa cases being male and a very wide confidence interval, the sex estimate is highly unstable and should not be interpreted as a reliable independent effect.

Adding age and sex increased the apparent AUC modestly; however, age and sex distributions also differed between the case and control groups, so the observed increment may partly reflect case-control selection rather than transportable biomarker value. The one-sided DeLong result and exploratory DCA were derived from the same development sample and should be interpreted cautiously. Any operating threshold requires prospective evaluation at the target prevalence and for a predefined intended use rather than reliance solely on the Youden index.

Exploratory findings for *PAX1* warrant cautious interpretation. The *PAX1*-only AUC was numerically higher than the *SOX1*-only AUC in this dataset (0.845 vs 0.828). *PAX1* was not added post hoc because it was designated as an exploratory comparator and adding a fourth predictor after inspection of the same small dataset would further increase overfitting; this design choice does not imply that *SOX1* is the superior biomarker. The observation that *SOX1* and *PAX1* methylation signals were elevated in other clinical groups is consistent with reports involving these genes in other tumor settings [22], 23] and suggest that the plasma signals may reflect a pan-neoplastic or field-effect signal rather than specificity for BCa. This characteristic may be cancer-associated or pan-neoplastic rather than specific for BCa. The current assay therefore cannot be considered a BCa-specific screening test.

Future studies should assess marker specificity across a broader spectrum of malignancies and benign inflammatory conditions and consider multi-marker panels (potentially integrating bladder-enriched methylation targets identified in large urinary studies) to improve tissue specificity and reduce false-positive findings [29], 30]. Any proposed tiered diagnostic strategy combining blood- and urine-based testing would require prospective evaluation before clinical use.

Several limitations should be acknowledged. The study was a retrospective, single-center, study with only 30 cases and 60 controls; no formal *a priori* sample-size calculation was performed, the combined model used three predictor parameters, and 29 of 30 cases were male. Coefficient instability, overfitting, and optimism are therefore important concerns. Second, all bootstrap, cross-validation, and calibration procedures reused the same 90 participants; there was no independent validation arm or cohort. Third, the two-gate case-control design, inclusion of 30 healthy controls, and fixed 1:2 case-control sampling fraction do not reproduce a consecutive hematuria population. Apparent AUC, sensitivity, and specificity may be affected by spectrum selection, while PPV, NPV, accuracy, calibration, and DCA are not directly transportable to a population with a different prevalence. Fourth, although available tumor stage and grade were reported descriptively, subgroup performance could not be analyzed reliably, and other pathological features were not consistently available. No head-to-head comparison with urine cytology, cystoscopy findings, or established urinary biomarkers was performed. Fifth, the single-centrifugation plasma workflow did not distinguish freely circulating, extracellular-vesicle-associated, or residual cell-derived DNA. Finally, the relative qMSP signal was normalized to a reference gene rather than measured as an absolute methylation percentage, so assay reproducibility and clinical cut-offs will require standardization and external analytical validation in accordance with published pre-analytical guidance for methylated cfDNA assays [27].

Accordingly, this study should be viewed as an initial feasibility and hypothesis-generating analysis. It provides internally derived estimates across discrimination, operating characteristics, calibration, and exploratory decision analysis, but it does not establish a validated test, generalizability, clinical utility, or an ability to reduce cystoscopy. Independent prospective multicenter studies in consecutive hematuria symptomatic or populations, with predefined clinical decision points and direct comparisons with current standards, are required.

## 5 Conclusions

A preliminary model combining plasma-derived *SOX1* methylation signal with age and sex was developed in a small selected case-control cohort. The combined model showed higher apparent discrimination than the *SOX1*-only model, but the estimates are vulnerable to overfitting, spectrum selection, and the absence of an independent validation cohort. The model is hypothesis-generating and not validated for clinical use; independent prospective evaluation in larger, prospective, and clinically representative cohorts is required.
